# Experimental perfect state transfer of an entangled photonic qubit

**DOI:** 10.1038/ncomms11339

**Published:** 2016-04-18

**Authors:** Robert J. Chapman, Matteo Santandrea, Zixin Huang, Giacomo Corrielli, Andrea Crespi, Man-Hong Yung, Roberto Osellame, Alberto Peruzzo

**Affiliations:** 1 Quantum Photonics Laboratory, School of Engineering, RMIT University, Melbourne, Victoria 3000, Australia; 2 School of Physics, The University of Sydney, Sydney, New South Wales 2006, Australia; 3 Istituto di Fotonica e Nanotecnologie, Consiglio Nazionale delle Ricerche, Piazza Leonardo da Vinci 32, Milano I-20133, Italy; 4 Dipartimento di Fisica, Politecnico di Milano, Piazza Leonardo da Vinci 32, Milano I-20133, Italy; 5 Department of Physics, South University of Science and Technology of China, Shenzhen 518055, China

## Abstract

The transfer of data is a fundamental task in information systems. Microprocessors contain dedicated data buses that transmit bits across different locations and implement sophisticated routing protocols. Transferring quantum information with high fidelity is a challenging task, due to the intrinsic fragility of quantum states. Here we report on the implementation of the perfect state transfer protocol applied to a photonic qubit entangled with another qubit at a different location. On a single device we perform three routing procedures on entangled states, preserving the encoded quantum state with an average fidelity of 97.1%, measuring in the coincidence basis. Our protocol extends the regular perfect state transfer by maintaining quantum information encoded in the polarization state of the photonic qubit. Our results demonstrate the key principle of perfect state transfer, opening a route towards data transfer for quantum computing systems.

## Abstract (web summary)

Transferring quantum information is a fundamental task, but doing so with high fidelity is a challenging task. Here, the authors implement the perfect state transfer protocol to a photonic qubit, entangled with a second one in a different location, across eleven coupled waveguides.

## Graphical abstract

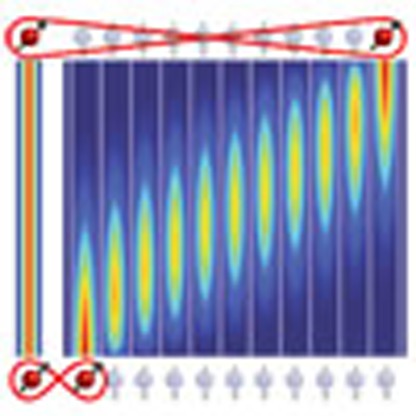

Transferring quantum information between locations without disrupting the encoded information *en route* is crucial for future quantum technologies^1^,^2^,^3^,^4^,^5^,^6^,^7^,^8^. Routing quantum information is necessary for communication between quantum processors, addressing single qubits in topological surface architectures, and for quantum memories as well as many other applications.

Coupling between stationary qubits and mobile qubits via cavity and circuit quantum electrodynamics has been an active area of research with promise for long-distance quantum communication^9^,^10^,^11^,^12^; however, coupling between different quantum information platforms is challenging as unwanted degrees of freedom lead to increased decoherence^13^. Quantum teleportation between distant qubits allows long-distance quantum communication via shared entangled states^14^,^15^,^16^,^17^; however, in most quantum information platforms this would again require coupling between stationary and mobile qubits. Physically relocating trapped ion qubits has also been demonstrated^18^,^19^, however, with additional decoherence incurred during transport.

By taking advantage of coupling between neighbouring qubits, it is possible to transport quantum information across a stationary lattice^2^. This has the benefits that one physical platform is being used and the lattice sites remain at fixed locations. The most basic method is to apply a series of SWAP operations between neighbouring sites such that, with enough iterations, the state of the first qubit is relocated to the last. This method requires a high level of active control on the coupling and is inherently weak as individual errors accumulate after each operation, leading to an exponential decay in fidelity as the number of operations increases^20^.

The perfect state transfer (PST) protocol utilizes an engineered but fixed coupled lattice. Quantum states are transferred between sites through Hamiltonian evolution for a specified time^2^,^3^,^4^,^5^,^6^,^7^. For a one-dimensional system with *N* sites, the state intially at site *n* is transferred to site *N*−*n*+1 with 100% probability without need for active control on the coupling^21^. PST can be performed on any quantum computing architecture where coupling between sites can be engineered, such as ion traps^18^ and quantum dots^22^. Figure 1 presents an illustration of the PST protocol. The encoded quantum state, initially at the first site, is recovered at the final site after a specific time. In the intermediate stages, the qubit is in a superposition across the lattice. Aside from qubit relocation, the PST framework can be applied to entangled W-state preparation^23^, state amplification^24^ and even quantum computation^25^,^26^,^27^,^28^,^29^.

To date, most research on PST has been theoretical^2^,^3^,^4^,^5^,^6^,^7^,^20^,^21^,^23^,^24^,^28^,^30^,^31^,^32^,^33^,^34^,^35^,^36^,^37^,^38^,^39^,^40^,^41^,^42^, with experiments^43^,^44^ being limited to demonstrations where no quantum information is transferred, and do not incorporate entanglement, often considered the defining feature of quantum mechanics^45^. Here, we present the implementation of a protocol that extends PST for relocating a polarization-encoded photonic qubit across a one-dimensional lattice, realized as an array of 11 evanescently coupled waveguides^46^,^47^,^48^. We show that the entanglement between a photon propagating through the PST waveguide array and another photon at a different location is preserved.

## Results

### PST Hamiltonian

**Figure 2:**
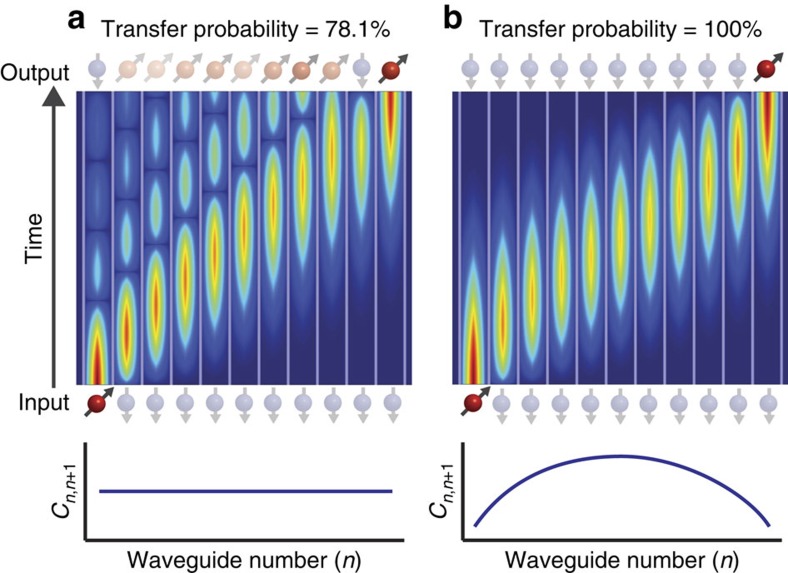
Propagation simulations with different coupling coefficient spectra. (**a**) A photon is injected into the first waveguide of an array of eleven coupled waveguides with the Hamiltonian in equation (1) and a uniform coupling coefficient spectrum. With the constraint that reflections off boundaries are not allowed, we calculate a maximum probability of transferring the photon to waveguide 11 of 78.1% (ref. 2). (**b**) A photon is injected into the first waveguide of an array of eleven coupled waveguides, this time with the coupling coefficient spectrum of equation (2). After evolution for a pre-determined time, the photon is received at waveguide 11 with 100% probability^3^,^4^,^5^,^6^,^7^.

The Hamiltonian for our system in the nearest-neighbour approximation is given by the tight-binding formalism


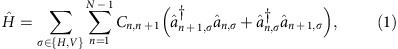


where *C*_*n*,*n*+1_ is the coupling coefficient between waveguides *n* and *n*+1, and 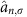 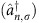 is the annihilation (creation) operator applied to waveguide *n* and polarization *σ* (horizontal or vertical). Hamiltonian evolution of a state 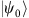 for a time *t* is calculated via the Schrödinger equation, giving the final state 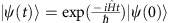 (ref. 49). Equation (1) is constructed of independent tight-binding Hamiltonians acting on each orthogonal polarization. This requires there to be no cross-talk terms 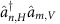 or 
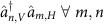
. The spectrum of coupling coefficients *C*_*n*,*n*+1_ is crucial for successful PST. Evolution of this Hamiltonian with a uniform coupling coefficient spectrum, equivalent to equally spaced waveguides, is not sufficient for PST with over three lattice sites as simulated in Fig. 2a. PST requires the coupling coefficient spectrum to follow the function


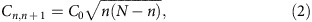


where *C*_0_ is a constant, *N* is the total number of lattice sites and evolution is for a specific time 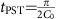 (refs 3, 4). This enables arbitrary-length PST as simulated in Fig. 2b for 11 sites. The coupling coefficient spectrum for each polarization must be equal and follow equation (2) for the qubit to be faithfully relocated and the polarization-encoded quantum information to be preserved. The distance between waveguides dictates the coupling coefficient; however, for planar systems, the coupling coefficient of each polarization will in general be unequal due to the waveguide birefringence. To achieve equal coupling between polarizations, the waveguide array is fabricated along a tilted plane in the substrate^50^. This is made possible by the unique three-dimensional capabilities of the femtosecond laser-writing technique (see Supplementary Note 1 for further fabrication and device details). We measure a total propagation loss of 1.8±0.2 dB; however, our figure of merit is how well preserved the polarization quantum state is after the transfer protocol. Therefore we calculate fidelity without loss. Ideally the PST protocol exhibits unit fidelity and efficiency, where the quantum state is reliably transferred and the encoded state is preserved. Due to loss in our experiment, we have less than unit efficiency; however, this loss is largely unrelated to the PST Hamiltonian in equation (1). Further optimizing the fabrication process could reduce the level of propagation loss (see Methods and Supplementary Note 2 for further details on loss).

**Figure 3:**
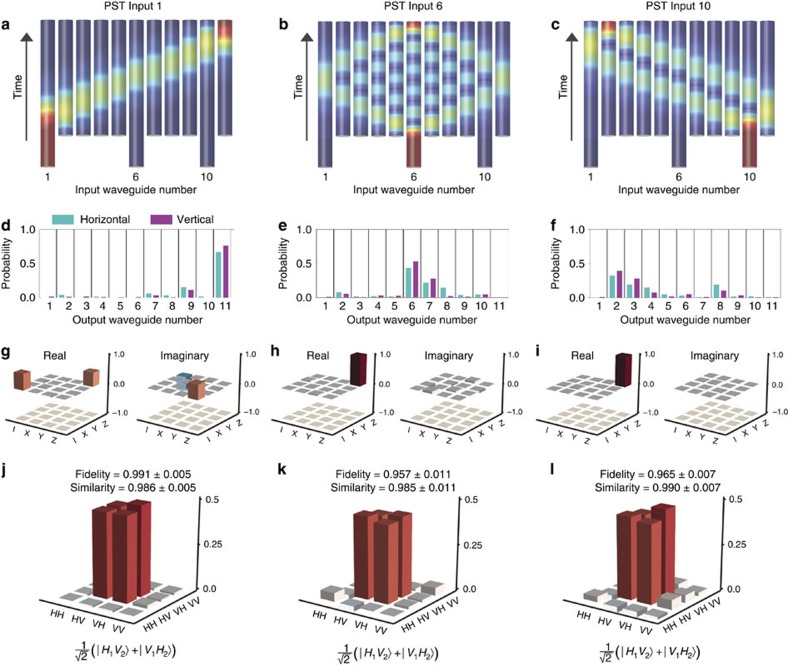
Experimental data from the characterization and performance of perfect state transfer waveguide array. (**a**–**c**) Propagation simulations showing the device implementation to enable specific waveguide input. (**d**–**f**) Output probability distributions for each input of the PST array for horizontally and vertically polarized laser light. (**g**–**i**) Quantum process matrix for each transfer in the PST array measured with single-photon quantum process tomography. (**j**–**l**) Two-photon quantum state tomography is performed after photon 1 of the polarization entangled Bell state 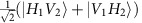 has been relocated. Results have had the small imaginary components removed for brevity.

We inject photons into waveguides 1, 6 and 10 of the array, which after time *t*_PST_ transfer to waveguides 11, 6 and 2, respectively. Figure 3a–c presents propagation simulations for each transfer. Input waveguides extend to the end of the device to allow selective injection.

### Transfer characterization

To characterize the coupling coefficient spectra, we inject horizontally and vertically polarized laser light at 808 nm into each input waveguide. Laser light is more robust to noise than single photons and we can monitor the output with a CCD camera to fast gather results. Using laser light at the same wavelength as our single photons will give an output intensity distribution equivalent to the output probability distribution for detecting single photons^46^. Ideally light injected into waveguide *n* will output the device only in waveguide *N*−*n*+1; however, this assumes an approximate model of nearest-neighbour coupling only. Taking into account coupling between further separated waveguides reduces the transfer probability. This decrease is greater for light injected closer to the centre of the array (see Supplementary Note 1). Figure 3d–f presents our measured output probability distribution for horizontally 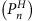 and vertically 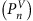 polarized laser light injected into each input waveguide, where *n* is the output waveguide number. Fidelity between the probability distributions for each polarization is given by 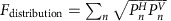. This fidelity is closely related to how similar the two coupling coefficient spectra are. We measure an average probability distribution fidelity for all transfers of 0.976±0.006 (see Supplementary Table 1 for all fidelity values). We encode quantum information in the polarization state of the photon and are interested in reliably relocating this qubit. We use a single optical fibre to capture photons from the designed output waveguide, which, in all cases, is the waveguide with the greatest output probability.

### Quantum process tomography

We perform quantum process tomography to understand the operation performed on the single-photon polarization state during each PST transfer. We inject single-photon states 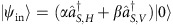 into each input waveguide *S*∈{1,6,10}, where *α* (*β*) is the probability amplitude of the horizontal (vertical) component of the photon and 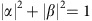. From quantum process tomography on the output polarization states, we can generate a process matrix *χ*_pol_ for each transfer^1^,^51^. We aim to perform the identity operation so that the quantum information encoded in the polarization can be recovered after relocation. We measure a polarization phase shift associated with each transfer. This phase shift can be compensated for with a local polarization rotation applied before injection. Figure 3g–i presents our measured process matrix for each transfer. Across all transfers we demonstrate an average fidelity of the polarization process including compensation to an identity of 0.982±0.003 (see Supplementary Note 3 for details of the compensation scheme and Supplementary Table 2 for all fidelities). Process fidelity is calculated as 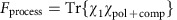 (ref. 52), where *χ*_1_ is the process matrix for the identity operation and *χ*_pol+comp_ is the combined polarization operation and compensation process matrix.

Ideally the output state for each transfer is 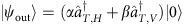, where *T*∈{11,6,2} and the probability amplitude of each polarization component remains equal to the input state. Our high-fidelity measurements on single-photon relocation demonstrate that we can route a polarization-encoded photonic qubit across our device and faithfully recover the encoded quantum information.

### Entangled state transfer

**Figure 4:**
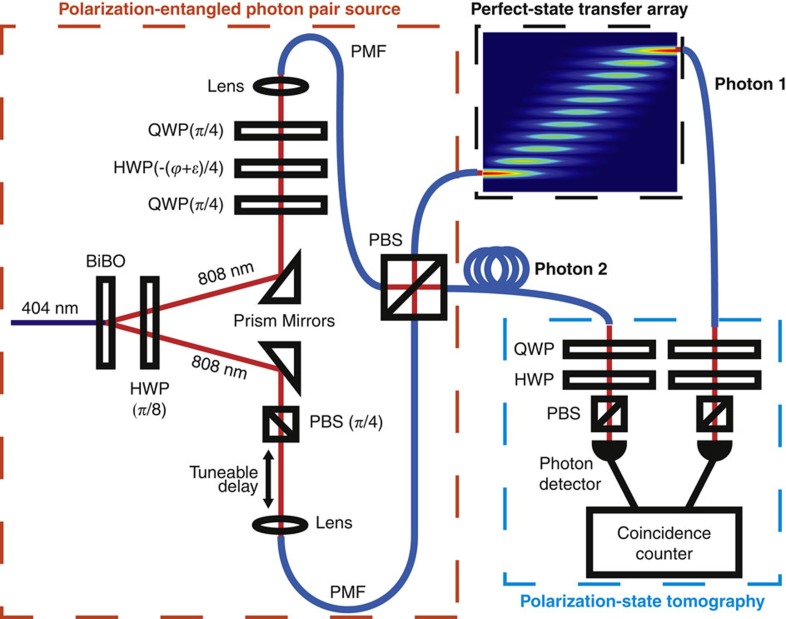
Experimental set-up. Polarization entangled photons are generated in free space before coupling into PMF. Photon 1 is injected into the perfect state transfer array, while photon 2 travels through PMF. Full two-qubit polarization tomography is performed on the output. See Methods for experimental set-up details.

Entanglement is likely to be a defining feature of quantum computing, and preserving entanglement is therefore critical to the success of any qubit relocation protocol. We prepare the Bell state 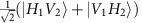 using the spontaneous parametric downconversion process. The polarization is controlled using rotatable half and quarter waveplates (HWPs and QWPs), and polarizing beam splitters (PBSs) as shown in Fig. 4 (ref. 53) (see Methods for details). This set-up prepares a general state 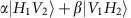 when measuring in coincidence, where 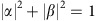. Photon 1 is injected into the waveguide array, while photon 2 propagates through polarization-maintaining fibre (PMF). In terms of waveguide occupancy, our input state is 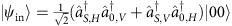 for each input waveguide *S*∈{1,6,10}, where 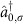 denotes the creation operator acting on polarization *σ* in PMF. Full two-qubit polarization tomography^54^ is performed on the output and the fidelity calculated as


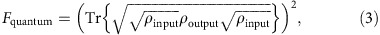


where *ρ*_output_ is the density matrix after the PST protocol has been applied and *ρ*_input_ is the density matrix after propagation through a reference straight waveguide^55^. After all qubit relocations we measure an average polarization state fidelity of 0.971±0.014. Fidelity is measured in the two-photon coincidence basis. This value is therefore the fidelity on the quantum state transferred without taking into account the loss (see Methods and Supplementary Note 2 for loss analysis). We can use the results from quantum process tomography to generate a characterized model of our device. We can now use this model to calculate the similarity between the predicted output state and our measured output state as


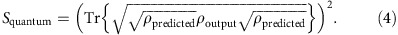


We calculate an average similarity of 0.987±0.014 across all transfers (see Supplementary Table 3 for all fidelities and similarities). Figure 3j–l presents our measured density matrix after each entangled state transfer.

Ideally, the output state for each transfer is 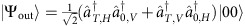, where *T*∈{11,6,2}. With high fidelity the probability amplitude of each component is preserved and the state remains almost pure. This result demonstrates that with our device we can relocate a polarization qubit between distant sites and preserve entanglement with another qubit at a different location. In principle our device could route qubits from any waveguide *n* to waveguide *N*−*n*+1. Quantum error correction protocols require sophisticated interconnection to access individual qubits for control and measurement within large, highly entangled surface code geometries^56^. PST is a clear gateway towards accessing qubits in such systems without disrupting quantum states and entanglement throughout the surface code.

### Decohered state transfer

**Figure 5:**
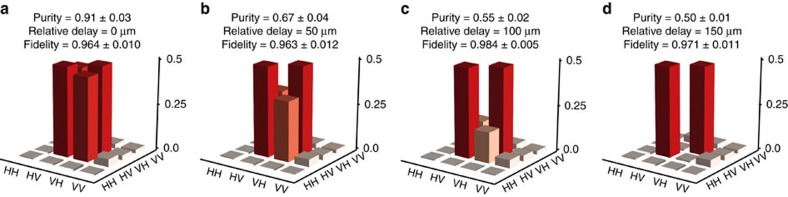
Perfect state transfer of entangled states with varying purity. Photon 1 of the state 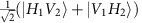 is injected into waveguide 1 of the PST array. A delay is applied to the vertical component to control the purity of the state. (**a**) Relative delay of 0 μm, (**b**) 50 μm, (**c**) 100 μm and (**d**) 150 μm. Results have had the small imaginary components removed for brevity.

Decoherence has applications in quantum simulation to emulate systems in nature^57^, and it is therefore important to note that this approach for relocating quantum information can be applied to states of any purity^3^. We prepare decohered states by introducing a time delay between the horizontal and vertical components of the polarization qubit. We implement this delay by extending one arm of the source, which reduces the overlap of the photons after they are both incident on the PBS, as shown in Fig. 4. This delay extends the state into a time-bin basis, which we trace over on measurement, leading to a mixed state. The purity of the state can be calculated as the convolution of the horizontal and vertical components with a time delay τ:


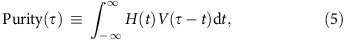


where *τ* is controlled by altering the path length of the vertical component of the state. *H*(*V*) is the horizontal (vertical) component of the photon. Figure 5 presents density matrices for PST from waveguide 1 to waveguide 11 applied to entangled states of varying purity. The injected states are recovered with an average fidelity of 0.971±0.019 and an average similarity of 0.978±0.019 (see Supplementary Table 4 for all values).

## Discussion

We have proposed and experimentally demonstrated a protocol for relocating a photonic qubit across eleven discrete sites, maintaining the quantum state with high fidelity and preserving entanglement with another qubit at a different location. We can aim to improve our fidelity by reducing next-nearest-neighbour coupling by further separating the waveguides and having a longer device. This would increase the contrast between nearest- and next-nearest-neighbour coupling to better fit the Hamiltonian in equation (1). A by-product of longer devices, however, is an increase in propagation loss. Depth-dependent spherical aberrations in the laser irradiation process may also affect the homogeneity of the three-dimensional waveguide array. Additional optics in the laser writing set-up could be employed to reduce this effect. Protocols for relocating quantum information across discrete sites are essential for future quantum technologies. Our protocol builds on the PST with extension to include an additional degree of freedom for encoding quantum information. This demonstration opens pathways towards faithful quantum state relocation in quantum computing systems.

## Methods

### Experimental set-up

Horizontally polarized photon pairs at 807.5 nm are generated via type 1 spontaneous parametric downconversion in a 1-mm-thick BiBO crystal, pumped by an 80-mW, 403.75-nm CW diode laser. Both photons are rotated into a diagonal state 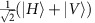 by a half waveplate (HWP) with fast axis at 22.5^°^ from vertical. One photon has a phase applied by two 45^°^ quarter waveplates (QWP) on either side of a HWP at *θ*°. The second photon has its diagonal state optimized with a PBS at ∼45^°^.

Each photon is collected in PMF and are incident on the two input faces of a fibre pigtailed PBS. When measuring in the coincidence basis, this post-selects the entangled state 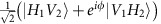, where 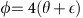 and 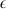 is the intrinsic phase applied by the whole system. The experimental set-up is illustrated in Fig. 4.

PMF is highly birefringent, resulting in full decoherence of the polarization state after ∼1 m of fibre giving a mixed state. To maintain polarization superposition over several metres of fibre, we use 90^°^ connections to ensure that both polarizations propagate through equal proportions of fast- and slow-axis fibre. Slight length differences between fibres and temperature variations mean the whole system applies a residual phase 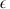 to the state, which can be compensated for in the source using the phase-controlling HWP.

Polarization state tomography combines statistics from projection measurements to generate the density matrix of a state. Single-photon rotations are applied by a QWP and HWP before a PBS. Single-qubit tomography requires four measurements and two-qubit tomography requires 16. Accidental counts are removed by taking each reading with and without an electronic delay. This helps reduce noise in our measurements.

### Photon count rate

In our experiment, we prepare polarization Bell states with a count rate of ∼2 × 10^3^ s^−1^. After the PST array we measure a count rate of ∼10^2^ s^−1^. The propagation loss of the array is only 1.8 dB. Most of the total loss (∼13 dB) is indeed due to mode mismatch between the waveguides and fibres, imperfect coupling, reflections at interfaces, and non-unit relocation efficiency. We integrate our measurements for 30 s to reduce the statistical noise due to the Poisson distribution of the photon count rate.

## Additional information

**How to cite this article:** Chapman, R. J. *et al.* Experimental perfect state transfer of an entangled photonic qubit. *Nat. Commun.* 7:11339 doi: 10.1038/ncomms11339 (2016).

## Supplementary Material

Supplementary Information Supplementary Tables 1-4 and Supplementary Notes 1-3

## Figures and Tables

**Figure 1:**
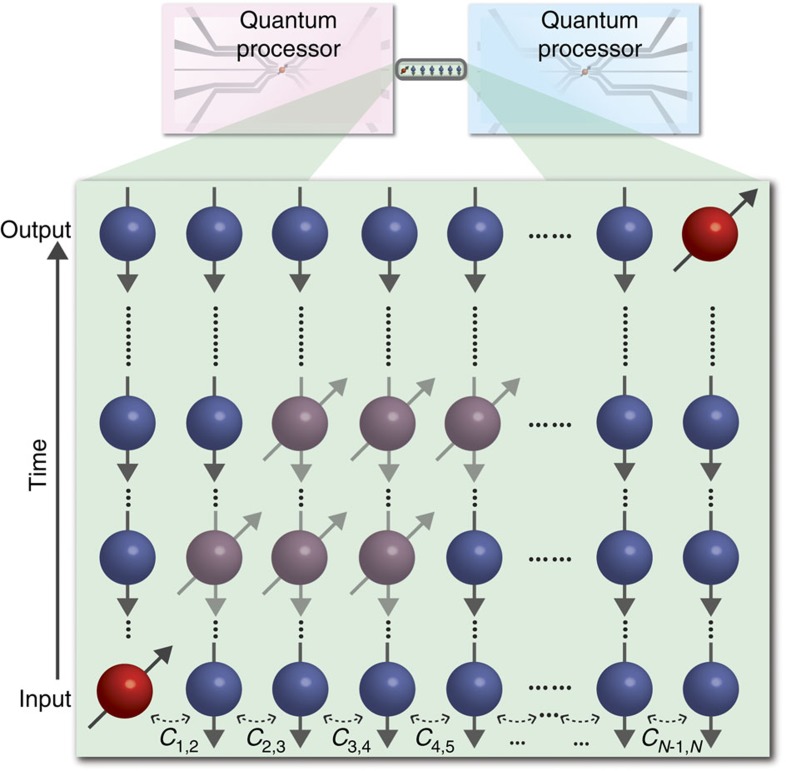
Illustration of a one-dimensional perfect state transfer lattice connecting two quantum processors. By engineering the Hamiltonian of a lattice, the state at the first site is transferred to the last site after a specific time. This Hamiltonian defines the perfect state transfer protocol^3^, which can be used for routing quantum information inside a quantum processor.
