# Supplementary material for: Experimental perfect state transfer of an entangled photonic qubit
Source: Nat Commun. 2016 Apr 18;7:11339. doi: 10.1038/ncomms11339 (PMC4837480; doi:10.1038/ncomms11339)
Supplement: Supplementary Information — Supplementary Tables 1-4 and Supplementary Notes 1-3 [file ncomms11339-s1.pdf]

Supplementary Information

Input Waveguide	Distribution Fidelity
1	0.956 ± 0.007
6	0.982 ± 0.005
10	0.989 ± 0.006

SUPPLEMENTARY TABLE 1. Output probability distribution fidelities. Horizontally and vertically polarised laser light is injected into each input waveguide of the PST array. The output probability distributions are measured with a CCD camera. The fidelity between the probability distribution of each polarisation is calculated for each transfer using equation 5 in Supplementary Note 1. This describes how similar coupling is between waveguides for each polarisation.

Input Waveguide	HWP (degrees)	Phase (degrees)	Fidelity to $\chi_{\mathbb{1}}$
1	0.438	-68.5	0.986 ± 0.002
6	0.349	44.7	0.975 ± 0.002
10	0.453	45.3	0.984 ± 0.002

SUPPLEMENTARY TABLE 2. Polarisation phase compensation. Each transfer imparts a polarisation phase and small linear rotation on the photonic qubit. This rotation is static for each transfer and thus can be characterised and compensated for. The compensation is achieved with a half-wave plate (HWP) that compensates for the small linear polarisation rotation, and a polarisation phase rotation. See figure 4 of the main text for the experimental setup. Fidelity is calculated between the measured quantum process and identity ($\chi_{\mathbb{1}}$) using equation 6 in Supplementary Note 2.

Input Waveguide	Fidelity	Similarity
1	0.991 ± 0.005	0.986 ± 0.005
6	0.957 ± 0.011	0.985 ± 0.011
10	0.965 ± 0.007	0.990 ± 0.007

SUPPLEMENTARY TABLE 3. Entangled state transfer. Photon 1 of a polarisation entangled Bell state is injected into the PST array, while photon 2 has its polarisation preserved in polarisation maintaining fibre. Two qubit polarisation state tomography is performed after photon 1 has propagated though the PST array and after photon 1 has propagated though a reference straight waveguide. The fidelity between these two measurements gives the fidelity of the PST operation on the polarisation state, which ideally would be the identity operator. Fidelity is calculated with equation 3 of the main text. The similarity (equation 4 of the main text) is calculated between the measured output state and the predicted state using a characterised model from quantum process tomography.

Delay (μm)	Fidelity	Similarity
0	0.964 ± 0.010	0.971 ± 0.010
50	0.963 ± 0.012	0.981 ± 0.012
100	0.984 ± 0.005	0.978 ± 0.005
150	0.971 ± 0.011	0.981 ± 0.011

SUPPLEMENTARY TABLE 4. Decohered state transfer. We demonstrate the transfer of decohered Bell states. Decohered states are prepared by introducing a delay between the two diagonally polarised photons before they are incident on both input faces of a polarising beam splitter (see figure 4 of the main text for experimental setup). When measuring in coincidence, this post-selects a Bell state. Introducing a delay between the photons, a Bell state with reduced coherence is prepared. With zero delay the state is a pure Bell state $\frac{1}{\sqrt{2}}(|HV\rangle + |VH\rangle)$. With a delay of $150\mu m$ (half the photon coherence length), the state is a mixture of the components $\frac{1}{2}(|HV\rangle\langle HV| + |VH\rangle\langle VH|)$.

SUPPLEMENTARY NOTE 1: TECHNICAL FABRICATION DETAILS

Waveguide fabrication Integrated photonic waveguides with single mode propagation at 808 nm are fabricated by focusing femtosecond laser pulses with an energy of 300 nJ/pulse, at the repetition rate of 1 MHz, in the bulk of a borosilicate substrate (Eagle2000, Corning) by means of a 20× microscope objective (NA = 0.45, Achroplan) and translating the sample at the constant speed of 40 mm/s.
The resulting waveguides exhibit relatively low propagation losses (0.8 dB/cm) and elliptical guided mode ($1/e^2$ diameters measured as 9.4 μm × 15.1 μm).

Design of the array The inter-waveguide distances are not uniform and are specially tailored in order to implement the correct couplings expressed by equation 2 in the main text, which allow the perfect state transfer protocol. The coupling between waveguides is a function of their separation d, according to the formula

$$C(d) = ae^{-bd}, \tag{1}$$

where a and b are constants whose values have been measured as $a = 3.6\,mm^{-1}$ and $b = 0.19\,\mu\text{m}^{-1}$. The distance d_n between waveguide n and waveguide $n+1$ of the array can be parametrised as follows:

$$d_n = d_{min} + \frac{1}{b}\log\left[\frac{1}{2}\sqrt{\frac{N^2-1}{n\,(N-n)}}\right], \tag{2}$$

where d_{min} is a free fabrication parameter that represents the minimum distance in the array and N (odd for this equation) is the total number of waveguides. With this parametrisation, the state transfer distance z_{PST} can be expressed as:

$$z_{PST} = \frac{\pi\sqrt{N^2-1}}{4C_{max}}, \tag{3}$$

where $C_{max} = C(d_{min})$. The choice of the values N and d_{min} must be done in order to minimise the array non-idealities such as propagation losses, array inhomogeneity and parasitic couplings between non adjacent sites. In our design we have chosen the values $N = 11$ and $d_{min} = 12\,\mu$m. Consequently, we fabricated 16 arrays with different lengths, spanning around the theoretical refocusing distance $z^{th}_{PST} = 23$ mm, ranging from 21.5 to 29 mm, and the best results were observed for an array length of 22.5 mm. In order to ensure that the inter-waveguide couplings for horizontally and vertically polarised light are the same, the arrays extend diagonally into the substrate, at an angle of $\approx 60°$. The central waveguide of each array, corresponding to waveguide 6, is situated 170 μm below the sample top surface. Finally, in order to couple light selectively in a given waveguide of the array, only waveguides identified by label 1, 6 and 10 reach the input facet of the devices, as depicted in Figures 3a-c of the main text.

Nearest Neighbour Approximation The PST protocol assumes the lattice has only nearest-neighbour interaction. The coupling between waveguides decays exponentially with distance and, therefore, higher-order coupling is observed, reducing the transfer success probability. Using a model with next-nearest-neighbour coupling, we calculate the expected transfer success from waveguide 1-11 to be 0.955, from waveguide 6-6 to be 0.604 and from waveguide 10-2 to be 0.901. This reduces the probability of transferring the photon to the correct output waveguide, however, the polarisation state is preserved as the coupling for both polarisations are very well matched.

Classical Characterisation In order to characterise the performances of the fabricated structures, we injected laser light in each of the available input ports and we measured the corresponding near field intensity profiles I_n^σ at the arrays output via a CCD camera and suitable imaging optics (label n indicates the waveguide number and σ the polarisation). We used laser light at 808 nm, which is very close to the wavelength employed in the single photon experiments. The output probability distribution P_n^σ for polarisation σ is then defined as:

$$P_n^\sigma = \frac{I_n^\sigma}{\sum_n I_n^\sigma}. \tag{4}$$

We repeated the experiment for both horizontally and vertically polarised light and the output probability distributions are shown in Figures 3d-f of the main text. We normalise the output intensity distribution and calculate the fidelity between the two polarisation probability distributions as:

$$F_{\text{distribution}} = \sum_n \sqrt{P_n^H P_n^V}. \tag{5}$$

Supplementary Table 1 gives the values of the distribution fidelities corresponding to each input waveguide.

SUPPLEMENTARY NOTE 2: PHOTON COUNT RATE AND LOSS

We generate polarisation Bell states at a rate of $\sim$ $2\text{x}10^3$ per second in coincidence. The propagation loss of the device is 1.8dB. We measure a total loss (including propagation loss) through a reference straight waveguide of $\sim$ 12dB. We measure an additional loss of $\sim$ 3dB when propagating through the array. This additional loss is due to non-unit relocation efficiency, largely a result of beyond nearest neighbour coupling. However, the majority of loss in our experiment is from coupling to and from the device; due to reflections at interfaces and mode mismatch between waveguides and fibres, as measured with the straight waveguide. We measure $\sim$ 80 photon pairs

in coincidence per second after the device.

SUPPLEMENTARY NOTE 3: QUANTUM PROCESS TOMOGRAPHY

Quantum process tomography (QPT) characterises an unknown quantum process by performing state tomography on a range of output states. We perform single qubit QPT using input states $|H\rangle$, $|V\rangle$, $|D\rangle = \frac{1}{\sqrt{2}}(|H\rangle + |V\rangle)$ and $|R\rangle = \frac{1}{\sqrt{2}}(|H\rangle + i|V\rangle)$. Each state is prepared through a straight waveguide, before switching to the PST array.

The output process matrices, χ, are shown in Figures 3g-i of the main text. From these process matrices we can determine a compensation scheme in terms of an HWP rotation and polarisation phase rotation. This is the same scheme as the state preparation and so the state preparation and compensation can be combined. The waveplate and phase angles of the pre-compensation scheme are given in Supplementary Table 2.

Process fidelity values describe how close the overall process (compensation and measured PST) is to identity on the polarisation. This is calculated as the trace distance between identity and the polarisation process

$$Tr\{\chi_{\mathbb{1}}\chi_{\text{pol+comp}}\}. \tag{6}$$

The compensation scheme gives a very good fidelity on how close to identity the whole operation will be. The reason it is not 100% is a result of some decoherence in the system, which cannot be compensated for with local unitary operations.
